# Effect of Dietary Vegetable Sources Rich in Unsaturated Fatty Acids on Milk Production, Composition, and Cheese Fatty Acid Profile in Sheep: A Meta-Analysis

**DOI:** 10.3389/fvets.2021.641364

**Published:** 2021-03-12

**Authors:** Einar Vargas-Bello-Pérez, Babak Darabighane, Florencia E. Miccoli, Pilar Gómez-Cortés, Manuel Gonzalez-Ronquillo, Marcello Mele

**Affiliations:** ^1^Department of Veterinary and Animal Sciences, Faculty of Health and Medical Sciences, University of Copenhagen, Copenhagen, Denmark; ^2^Department of Animal Science, University of Mohaghegh Ardabili, Ardabil, Iran; ^3^Facultad de Ciencias Agrarias, Universidad Nacional de Lomas de Zamora (UNLZ), Buenos Aires, Argentina; ^4^Departamento de Producción Animal, Facultad de Agronomía, Universidad de Buenos Aires (UBA), Buenos Aires, Argentina; ^5^Departamento de Bioactividad y Análisis de Alimentos, Instituto de Investigación en Ciencias de la Alimentación (CSIC-UAM), Universidad Autónoma de Madrid, Madrid, Spain; ^6^Facultad de Medicina Veterinaria y Zootecnia, Universidad Autónoma del Estado de México, Toluca, Mexico; ^7^Dipartimento di Scienze Agrarie, Alimentari e Agro-ambientali, Università di Pisa, Pisa, Italy

**Keywords:** biohydrogenation, cheese, fatty acid, lipid, dairy

## Abstract

A meta-analysis was conducted to analyze the effects of different dietary vegetable sources rich in unsaturated FA (UFA) on sheep cheese FA profile. This study also quantified the overall effect of feeding sheep with vegetable sources rich in UFA (linseed, flaxseed, sunflower seed, canola, olive oil, bran oil, and olive cake), on milk yield (MY) and milk composition. A literature search was conducted to identify papers published from 2000 to 2019. Effect size for all parameters was calculated as standardized mean difference. Heterogeneity was determined using *I*^2^ statistic, while meta-regression was used to examine factors influencing heterogeneity. Effect size was not significant for MY, milk fat percentage (MFP), and milk protein percentage (MPP). Dietary inclusion of vegetable sources rich in UFA decreased the effect size for C12:0, C14:0, and C16:0 and increased the effect size for C18:0, C18:1 t-11, C18:1 c-9, C18:2 c-9, t-11, C18:2 n-6, and C18:3 n-3. Heterogeneity was significant for MY, MFP, MPP, and overall cheese FA profile. Meta-regression revealed days in milk as a contributing factor to the heterogeneity observed in MFP and MPP. Meta-regression showed that ripening time is one of the factors affecting cheese FA profile heterogeneity while the type of feeding system(preserved roughages vs. pasture) had no effect on heterogeneity. Overall, inclusion of dietary vegetable sources rich in UFA in sheep diets would be an effective nutritional strategy to decrease saturated FA and increase polyunsaturated FA contents in cheeses without detrimental effects on MY, MFF, and MPP.

## Introduction

Dairy sheep and goats account for ~3.5% of the world's milk production (1). Dairy sheep are concentrated around the European Mediterranean and Black Sea regions, where their dairy products are included in the regular diet of the population and would be of interest from a nutritional standpoint (2).

Today, many consumers are aware about the relation between diet and health (3). In this sense, saturated fatty acids (FA), which are detected in significant amounts in dairy fat, have been of the greatest concern. Although increasing scientific evidences are reporting no beneficial effects of reducing saturated FA intake on cardiovascular disease (4), usually consumers relate this group of fats to health-related issues (5). In terms of costs, time, and responses, in dairy ruminants, the most effective strategy to modulate milk FA toward a healthier profile for human consumption is through dietary changes (6, 7).

In the specific case of milk and cheese from sheep, several feedstuffs have been used to increase contents of specific FA such as vaccenic, rumenic, and α-linolenic acids with potential positive effects on human health (8). Over the past two decades, different lipid supplements such as flaxseed (9), sunflower seed (10–12), linseed (12), extruded linseed (13–16), canola oil (17), olive oil (18, 19), and olive cake (20) have been evaluated in sheep diets to increase contents of bioactive FA in sheep cheese.

A meta-analysis is an approach that combines results of different studies and compile them statistically (21). Also, a meta-analysis reports the mean effect size of an intervention factor and also investigates the between-study variability or heterogeneity of treatment effects (22). One objective of this study was to analyze the effects of different dietary vegetable sources rich in unsaturated FA (UFA) on sheep cheese FA profile. A second objective was to quantify the overall effect of feeding sheep with vegetable sources rich in UFA on milk yield (MY) and milk composition. Data from this study will be helpful to quantitatively summarize the impact of dietary factors on MY, milk components, and cheese FA profile.

## Materials and Methods

### Search of the Published Literature

A comprehensive search of the literature published in English from 2000 to 2019 was conducted in order to identify experiments focused on analyzing the effects of dietary vegetable sources rich in unsaturated FA (UFA) on FA profiles of sheep cheeses. The literature search included two search engines, the ISI Web of Knowledge (http://wokinfo.com) and Google Scholar (http://scholar.google.com). The keywords used to search relevant studies included oilseed, fatty acids, and cheese and dairy ewe. For Google Scholar, several thousand hits were collected, and results were sorted in order of relevance. The screening of papers stopped after at least 50 records after the last relevant record was identified. There were no restrictions on the selection of journals in terms of impact factor and quartile ranking.

### Inclusion and Exclusion Criteria

**Figure 1:**
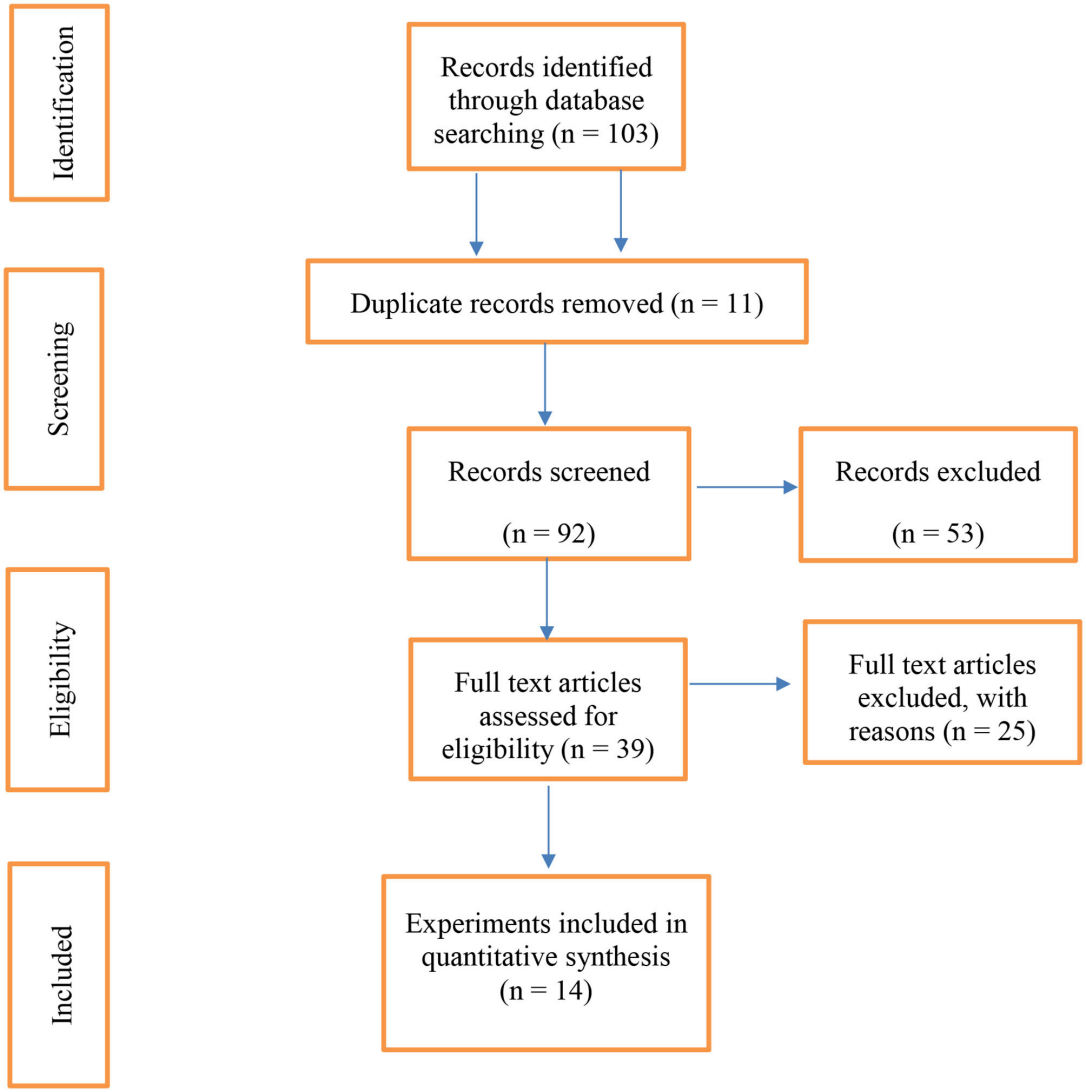
The PRISMA flow diagram of the systematic review from initial search and screening to final selection of publications to be included in the meta-analysis.

Figure 1 shows a PRISMA flow diagram (23) of the data collected for the meta-analysis. Out of 103 published articles, duplicate articles (*n* = 11), review articles (*n* = 30), articles related to the effect of dietary vegetable sources rich in UFA in other livestock species (cattle and goats; *n* = 19), and articles related to *in vitro* experiments (*n* = 4) were excluded. Of the remaining 39 articles, those that only reported FA profiles of milk, yogurt, or adipose tissue (*n* = 24) or lacked a control group (*n* = 1) were excluded. Therefore, the 14 articles identified for this meta-analysis had the main criterion (the effects of dietary vegetable sources rich in UFA on sheep cheese FA profile). Two reviewers (FEM and BD) screened all available articles against the inclusion and exclusion criteria. Disagreements between reviewers were resolved at all stages by consultation with a third reviewer (EVBP). A list of the experiments included in the meta-analysis is depicted in Table 1.

**Table 1:** Summary of papers used for the meta-analysis.

**References**	**No. of comparisons**	**Country**	**Breed**	**Type of vegetable sources rich in unsaturated fatty acids**	**Amount of vegetable sources rich in unsaturated fatty acids**	**Ripening times (days)**	**Forage basis**	**Evaluated variables^***a***^**
Addis et al. (12)	2	Italy	Sarda	Sunflower seeds; Linseeds	^1^40 g/kg DM sunflower seeds + 50 g/kg DM g/d of linseeds; 12 g/kg DM of sunflower seeds and 100 g/kg DM of linseed	30	Pasture	C18:1 t-11; C18:2 c-9, t-11; C18:2 n-6; C18:3 n-3; SFA; PUFA
Bodas et al. (18)	3	Spain	Churra	Olive oil; Soybean oil; Linseed oil	30 g/kg DM; 30 g/kg DM; 30 g/kg DM	60	Preserved roughages	MY; MFP; MPP; MLP; C12:0; C14:0; C16:0; C18:0; C18:1 t-11; C18:1 c-9; C18:2 c-9, t-11; C18: 2 n-6; C18:3 n-3; SFA; MUFA; PUFA
Branciari et al. (14)	2	Italy	Sarda	Extruded linseed	^1^30 g/kg DM; 60 g/kg DM	60	Pasture	C18:2 c-9, t-11; SFA; MUFA; PUFA
Branciari et al. (13)	2	Italy	Sarda	Extruded linseed	^1^30 g/kg DM; 60 g/kg DM	60	Pasture	C12:0; C14:0; C16:0; C18:0; C18:1 t-11; C18:1 c-9; C18:2 c-9, t-11; C18:2 n-6; C18:3 n-3; SFA; MUFA; PUFA
Fusaro et al. (24)	1	Italy	Comisana	Extruded linseed	98 g/kg DM	1	Preserved roughages	SFA; MUFA; PUFA
Gómez-Cortés et al. (25)	2	Spain	Manchega	Extruded linseed	60 g/kg DM; 120 g/kg DM	90	Preserved roughages	MY; MFP; MPP; MLP; C12:0; C14:0; C16:0; C18:0; C18:1 t-11; C18:1 c-9; C18:2 c-9, t-11; C18:2 n-6; C18:3 n-3; SFA; MUFA; PUFA
Mele et al. (15)	1	Italy	Sarda	Extruded linseed	^1^95 g/kg DM	60	Preserved roughages	C12; C14; C16; C18; C18:1 t-11; C18:1 c-9; C18:2 c-9, t-11; C18:2 n-6; C18:3 n-3
Mughetti et al. (16)	2	Italy	Sarda	Extruded linseed	100 g/kg DM; 200 g/kg DM	60	Pasture	MY; MFP; MPP; MLP; C12:0; C14:0; C16:0; C18:0; C18:1 t-11; C18:1 c-9; C18:2 c-9, t-11; C18:2 n-6; C18:3 n-3; SFA; MUFA; PUFA
Nguyen et al. (17)	4	Australia	Awassi and Awassi × East Friesian	Canola oil; Rice bran oil; Flaxseed oil; Sunflower oil	Wheat-based pellets + 50 mL/kg DM	120	Pasture	C12:0; C14:0; C16:0; C18:0; C18:1 c-9; C18:2 c-9, t-11; C18:2 n-6; C18:3 n-3; SFA; MUFA; PUFA
Vargas-Bello-Pérez et al. (19)	2	Chile	Crossbreed between Finnish Landrace, Border Leicester, Poll Dorset and Merino Precoz	Olive oil	36 g/kg DM; 88 g/kg DM	60	Preserved roughages	MY; MFP; MPP; C12:0; C14:0; C16:0; C18:0; C18:1 t-11; C18:1 c-9; C18:2 n-6; C18:3 n-3
Vargas-Bello-Pérez et al. (20)	2	Chile	Crossbreed between Finnish Landrace, Border Leicester, Poll Dorset and Merino Precoz	Olive cake	98 g/kg DM; 244 g/kg DM	60	Preserved roughages	MFP; MPP; C12:0; C14:0; C16:0; C18:0; C18:1 t-11; C18:1 c-9; C18:2 n-6; C18:3 n-3; SFA; MUFA; PUFA
Zhang et al. (9)	3	Canada	Suffolk × East Friesian	Flaxseed	90 g/kg DM; 180 g/kg DM; 260 g/kg DM	1	Preserved roughages	MY; MFP; MPP; MLP; C12:0; C14:0; C16:0; C18:0; C18:1 t-11; C18:1 c-9; C18:2 c-9, t-11; C18:2 n-6; C18:3 n-3; SFA; MUFA; PUFA
Zhang et al. (11)	2	Canada	East Friesian × Lacunae	Flaxseed; Sunflower seed;	67 g/kg DM; 59 g/kg DM	1	Preserved roughages	MY; MFP; MPP; MLP; C12:0; C14:0; C16:0; C18:0; C18:1 t-11; C18:1 c-9; C18:2 c-9, t-11; C18:2 n-6; C18:3 n-3; SFA; MUFA; PUFA
Zhang et al. (10)	3	Canada	Dorset	Canola seed; Sunflower seed; Flaxseed	73 g/kg DM; 66 g/kg DM; 80 g/kg DM	1	Preserved roughages	MY; MFP; MPP; MLP; C12:0; C14:0; C16:0; C18:0; C18:1 c-9; C18:2 c-9, t-11; C18:2 n-6; C18:3 n-3; SFA

*MY, milk yield; MFP, milk fat percentage; MPP, milk protein percentage; MLP, milk lactose percentage; SFA, saturated fatty acid; MUFA, monounsaturated fatty acid; PUFA, polyunsaturated fatty acid*.

### Data Extraction

Data extracted from each study included authors' names, and year of publication, MY (g per day), milk fat percentage (MFP), milk protein percentage (MPP), milk lactose percentage (MLP), and cheese FA profile (g/100 g). Data including country, breed of sheep, type of vegetable sources rich in UFA, amount of vegetable sources rich in UFA, forage basis (pasture or preserved roughages), number of animals in experimental groups (control and treatments), and standard error were also extracted. The standard deviation (SD) was recorded as the measure of variance. If SD was not reported in studies, it was calculated by multiplying the reported SE of means by the square root of the sample size.

One of the limitations in this meta-analysis was the non-reporting of some data related to chemical composition of diet and fatty acid profiles from experimental diets. However, we tried to consider all available information [days in milk (DIM) and ripening time] useful as a variable to perform meta-regression for MY, milk composition, and cheese FA profiles.

Data were transferred to Excel spreadsheets (version 2016, Microsoft Corp., Redmond, WA) and reviewed by two researchers to assure that data collected was accurately transcribed from the manuscripts into the spreadsheets before statistical analyses.

### Statistical Analysis

#### Descriptive Statistics

Descriptive statistics for all parameters (MY, MFP, MPP, MLP, and cheese FA profile) were performed using Excel spreadsheets (version 2016, Microsoft Corp., Redmond, WA).

#### Effect Size and Forest Plots

Statistical analysis was performed using Comprehensive Meta-Analysis (CMA) software version 3 (Biostat, USA) to calculate effect size for MY, milk components (fat, protein and lactose), and cheese fatty acid profile in terms of standardized means difference (SMD) at a 95% confidence interval. The SMD indicates mean difference between treatment and control groups standardized based on SD of treatment and control groups (26). The SMD is calculated using the following formula:

$$\begin{array}{l} SMD=\frac{{\bar{\text{x}}}_{\text{e}}-{\bar{\text{x}}}_{\text{c}}}{{\text{S}}_{\text{p}}} \end{array}$$

where ${\bar{x}}_{e}$ is the experimental group mean, ${\bar{x}}_{c}$ is the control group mean, and *S*_*p*_ is the pooled SD (27).

In addition to calculating the SMD, for each outcome, the raw mean difference (RMD) was calculated with a 95% confidence interval. The RMD is the difference between the control and treatment groups. Calculating RMD allows expression of the effect size with the same unit as the measurement. A random-effect model was adopted for the meta-analysis, which has an underlying assumption that the distribution of effects exists, resulting in heterogeneity among study results (26). Significance of effect size estimates (SMD and RMD) was declared at *p* ≤ 0.05.

Forest plots were constructed to evaluate the effects of dietary vegetable sources rich in UFA on MY, MFP, C18:2 c-9, t-11, and polyunsaturated FA (PUFA) in cheese. Effect size for forest plot was the SMD at 95% confidence interval using the random-effect model.

#### Heterogeneity

Statistical heterogeneity refers to the true effects in each study not being identical (21). The existence of heterogeneity reflects underlying differences in clinical diversity of the herds, differences in study design, and statistical variation (27). Identifying the presence and sources of the heterogeneity improves understanding of the responses to the interventions used. Heterogeneity of results among the trials was quantified using the *I*^2^ statistic (27).

$$\begin{array}{l} I^{2}\left(\%\right)=\frac{Q-\left(k-1\right)}{Q}\;\times 100 \end{array}$$

where *Q* is the χ2 heterogeneity statistic and *k* is the number of trials. An *I*^2^ value between 0 and 40% might not be important, 30–60% may represent moderate heterogeneity, 50–90% might represent substantial heterogeneity, and 75–100% might represent considerable heterogeneity (28).

#### Meta-regression

Meta-regression analyses were used to explore the source of heterogeneity of response, using the individual SMD for each study comparison as the outcome and the associated SE as the measure of variance. In this study, meta-regression analysis was used to evaluate heterogeneous sources for parameters whose *I*^2^ is more than 50%. Meta-regression was estimated through the method of moments also known as DerSimonian and Laird method. This method of estimating the variance between studies is well-established (26).

In this study, the DIM variable was used as a covariate for data related to MY and composition; ripening time and type of feeding system (preserved roughages vs. pasture) were used as covariates for the cheese FA profile. As mentioned before, some studies did not report complete data including the chemical composition of diet and dietary FA profiles. This limitation did not allow the use of some parameters as covariates in meta-regression due to the scarcity of available data.

#### Publication Bias

Although a meta-analysis will yield a mathematically accurate synthesis of the studies included in the analysis, if these studies are a biased sample of all relevant studies, then the mean effect computed by the meta-analysis will reflect this bias. This issue is generally known as publication bias. Egger's linear regression asymmetry was used to examine the presence of publication bias. When significant (*P* < 0.10) bias was detected, the trim-and-fill method (29) was applied to find the number of missing observations. Funnel plots were used to present asymmetry. This technique indicates symmetric distribution of effect sizes around the true effect size if it is assumed that no publication bias exists, that is, the most extreme results have not been published. In this study, the trim-and-fill method and funnel plot were used for C18:2 c-9, t-11, and PUFA.

## Results

### Data Overview

Table 1 shows the selected papers and the data extracted for the meta-analysis. For the meta-analysis, 14 papers (studies) were included. Eight studies were performed in Europe (Italy and Spain), three studies in Canada, two studies in Chile, and one study in Australia. Sarda sheep were used in most studies (12–16).

In terms of feeding system, studies were divided into two categories: diets where the forage consisted of preserved roughages and diets where the forage was based on pastures. In 9 studies, the feeding regimen was based on preserved roughages (15, 18–20, 24, 25) and the rest were pasture-based systems (12–14, 16, 17).

The vegetable sources rich in UFA varied across studies: linseed (12–16, 18, 24, 25), flaxseed (9–11, 17), sunflower seed (10–12, 17), canola (9, 17), olive oil (18, 19), bran oil (17), and olive cake (20).

In 10 studies out of 14, the amount of lipid supplementation was expressed as g per kg of dry matter (DM) intake. When the articles reported the datum expressed as percentage of concentrate intake (13–15) or as g of supplement per day (12), the amount of lipid supplementation per kg of expected DM intake was estimated on the basis of the amount of concentrate administrated and of the expected DM intake. The expected DM intake was calculated by using the information reported about the breed and the productive level of the animals employed in the studies. The amount of extruded linseed administrated ranged between 3 and 12% of DM intake, whereas the amount of crude linseed ranged from 6.7 to 26%. Sunflower seed supplementation ranged between 5.9 and 6.7% of DM intake.

Pure oil was administrated in 3 experiments out of 14, using olive oil, soybean oil, linseed oil, canola oil, rice bran oil, and sunflower oil. Olive oil was tested in two studies, and the amount of supplementation ranged between 30 and 88 g/kg of DM. As regard the other vegetable oils, the range of supplementation was between 30 and 45 g/kg of DM (Table 1). The ripening time of cheese ranged from 1 to 120 days. From all studies, MY from fat-supplemented rations was 1,254 g/d and in the control (unsupplemented fat rations) was 1,219 g/d (Table 2).

**Table 2:** Descriptive statistics of data used in meta-analysis.

**Variable^**a**^**	**Mean**		**SD**		**Min**		**Max**	
	**S^**b**^**	**U^**b**^**	**S**	**U**	**S**	**U**	**S**	**U**
**Productive parameters**								
MY (g/d)	1,254	1,219	704	733	374	382	2,700	2,600
MFP (%)	6.70	6.39	2.12	1.96	1.90	2.00	9.55	8.43
MPP (%)	5.26	5.25	0.67	0.74	4.40	4.20	6.60	6.39
MLP (%)	4.69	4.71	0.27	0.28	4.27	4.28	5.05	5.10
**Fatty acids (g/100 g)**								
C12:0	3.15	4.15	1.16	1.68	1.02	0.79	5.17	5.72
C14:0	9.57	10.7	1.64	1.60	6.07	8.53	13.7	14.7
C16:0	23.5	27.0	5.27	4.36	16.0	20.5	34.2	35.8
C18:0	12.3	10.6	4.04	2.44	8.44	6.58	23.0	14.3
C18:1 t-11	4.35	2.05	2.17	0.91	1.50	0.90	10.5	3.72
C18:1 c-9	19.0	16.6	6.66	6.47	0.39	0.33	27.8	24.5
C18:2 c-9, t-11	1.65	0.88	0.61	0.29	0.72	0.43	2.71	1.55
C18:2 n-6	2.66	2.29	0.82	0.51	1.46	1.52	4.78	3.26
C18:3 n-3	1.13	0.73	0.58	0.21	0.31	0.43	2.10	1.18
ΣSFA	62.8	67.9	3.93	6.32	56.8	59.0	74.7	78.0
ΣMUFA	27.7	25.6	4.81	6.61	18.8	18.1	35.9	35.1
ΣPUFA	6.84	4.95	1.70	0.97	2.85	3.85	9.60	6.64

a *MY, milk yield; MFP, milk fat percentage; MPP, milk protein percentage; MLP, milk lactose percentage; SFA, saturated fatty acid; MUFA, monounsaturated fatty acid; PUFA, polyunsaturated fatty acid*.

b *S, fat-supplemented rations; U, nonfat-supplemented rations*.

### Milk Yield and Milk Components

**Figure 2:**
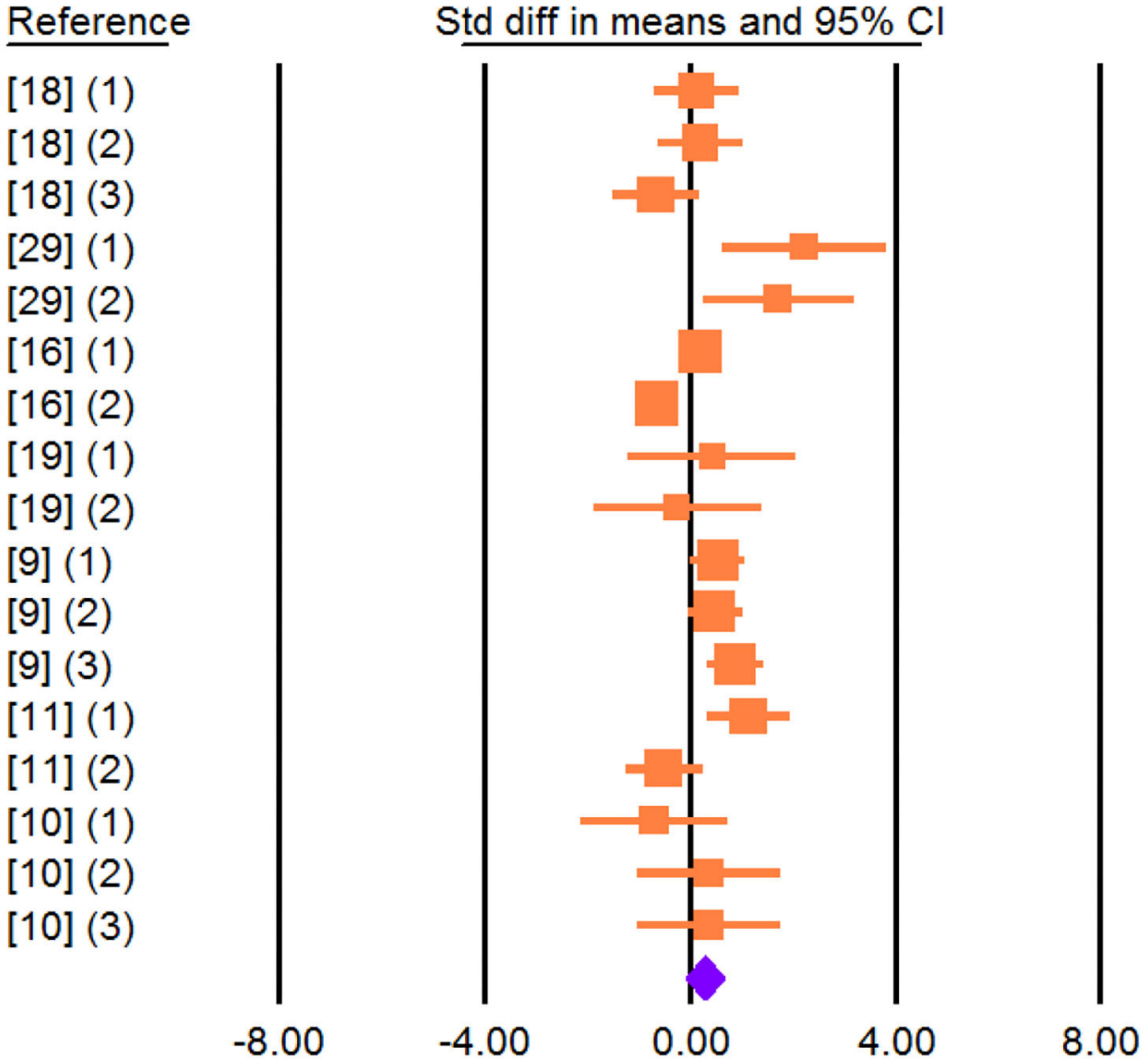
Forest plot of the effect of dietary vegetable sources rich in unsaturated fatty acids on milk yield in dairy ewe based on standardized mean differences (Std. diff in means). The diamond at the bottom indicates the mean effect size, calculated according to a random-effect model. The size of the squares illustrates the weight of each study relative to the mean effect size. Smaller squares represent less weight. The horizontal bars represent the 95% confidence intervals for the study.

Effect size, heterogeneity, and publication bias for the effect of dietary vegetable sources rich in UFA on MY and milk composition in dairy ewe are shown in Table 3. Inclusion of vegetable sources rich in UFA in the diet of dairy ewe resulted in non-significant increase in effect size for MY (SMD = 0.266; *P* = 0.142; Figure 2).

**Table 3:** Effect size, heterogeneity, and publication bias for the effect of dietary vegetable sources rich in unsaturated fatty acids on milk yield and milk composition in dairy ewe.

		**SMD**^****a****^ **(95% Cl**^****b****^**)**		**Heterogeneity**		**RMD^**c**^ (95% Cl)**	**Publication bias**
**Outcomes**	**No. of comparisons**	**Random effect**	***P*-value**	***I*^**2**^**	***P*-value**	**Random effect**	**Egger**
Milk yield	17	0.266	0.142	77.075	<0.001	22.449	0.151
		(−0.089, 0.622)				(−14.709, 59.607)	
Milk fat %	19	0.142	0.595	88.445	<0.001	0.237	0.571
		(−0.381, 0.664)				(0.127, 0.348)	
Milk protein %	19	−0.246	0.358	88.463	<0.001	0.028	0.129
		(−0.770, 0.278)				(−0.016, 0.072)	
Milk lactose %	15	0.426	0.049	83.502	<0.001	0.038	0.866
		(0.002, 0.850)				(0.010, 0.067)	

a SMD, standardized mean difference;

b Cl, confidence interval;

c *RMD, raw mean difference*.

**Figure 3:**
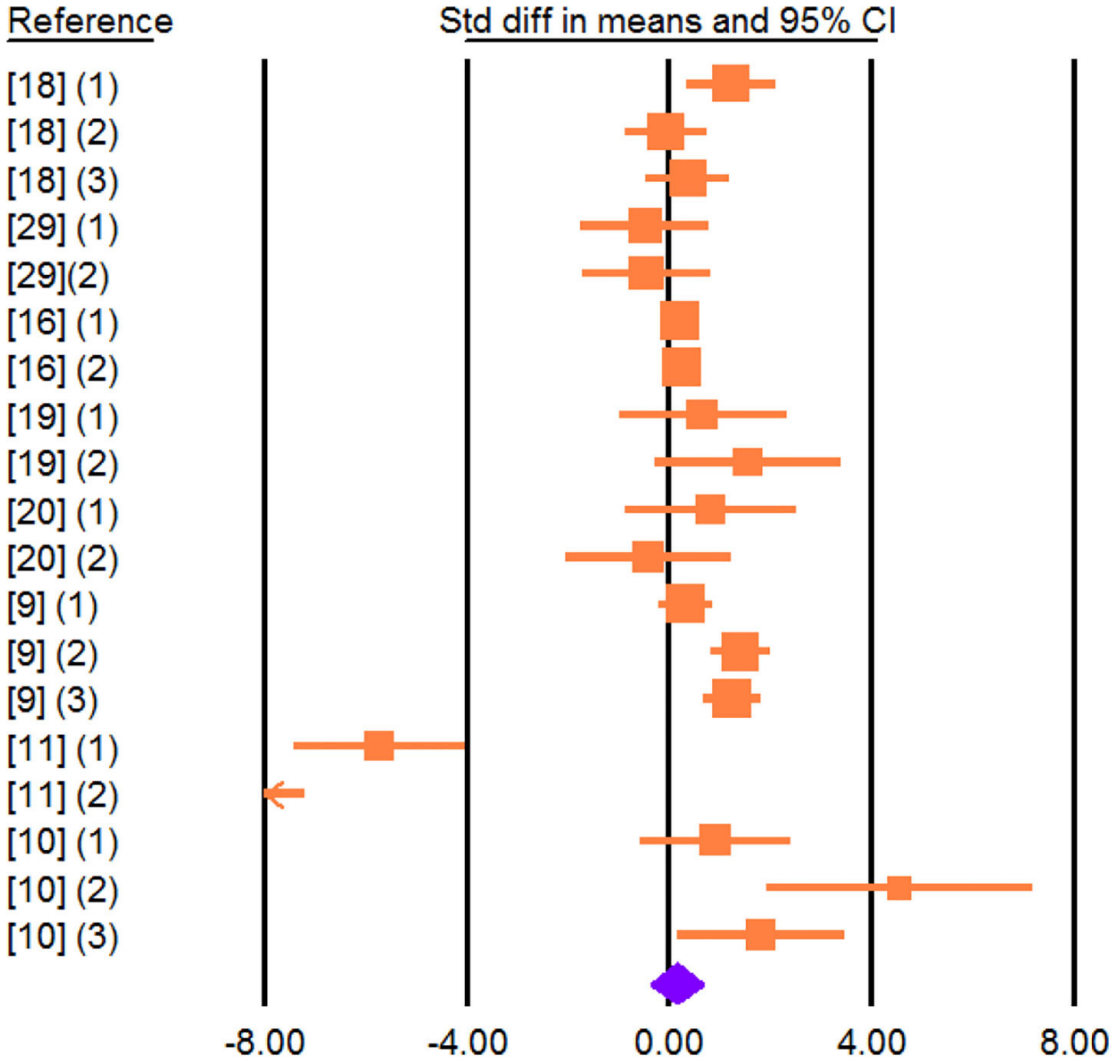
Forest plot of the effect of dietary vegetable sources rich in unsaturated fatty acids on milk fat percentage in dairy ewe based on standardized mean differences (Std. diff in means). The diamond at the bottom indicates the mean effect size, calculated according to a random-effect model. The size of the squares illustrates the weight of each study relative to the mean effect size. Smaller squares represent less weight. The horizontal bars represent the 95% confidence intervals for the study.

Effect size was non-significant for MFP (SMD = 0.142; *P* = 0.595; Figure 3) and MPP (SMD = −0.246; *P* = 0.358) but increased for MLP (SMD = 0.426; *P* = 0.049).

Heterogeneity was significant for MY, MFP, MPP, and MLP (*I*^2^, Table 3). Meta-regression revealed DIM as a contributing factor to the heterogeneity observed in MFP and MPP (Tables 3, 4). These results showed that with increasing DIM, the MFP and MPP increase. Egger's test found no publication bias for MY and milk components (*P* < 0.1; Table 3).

**Table 4:** Summary of meta–regression analysis output for milk yield and composition that were influenced by days in milk.

	**Item**	**Coefficient**	**95% CI^***a***^**	***P*-value**
Milk yield	Intercept	1.306	−0.007, 2.620	0.051
	Days in milk	−0.025	−0.056, 0.005	0.107
Milk fat %	Intercept	−2.454	−4.102, −0.807	0.003
	Days in milk	0.064	0.025, 0.102	0.001
Milk %	Intercept	−2.816	−4.636, −0.997	0.002
	Days in milk	0.062	0.020, 0.105	0.003
Milk lactose %	Intercept	1.601	0.229, 2.973	0.022
	Days in milk	−0.029	−0.061, 0.003	0.073

a *Cl, confidence interval*.

### Cheese Fatty Acid Profile

Table 5 presents effect size, heterogeneity, and publication bias for the effect of dietary vegetable sources rich in UFA on cheese FA profiles in dairy ewe. Inclusion of vegetable sources rich in UFA in the diet of dairy ewe significantly decreased C12:0, C14:0, and C16:0 contents in cheese. Adding vegetable sources rich in UFA in the diet significantly increased C18:0 (SMD = 2.949; *P* < 0.001).

**Table 5:** Effect size, heterogeneity, and publication bias for the effect of dietary vegetable sources rich in unsaturated fatty acids on cheese fatty acid profiles.

		**SMD**^******b******^ **(95% CI**^**c**^**)**		**Heterogeneity**		**RMD^**d**^ (95% CI)**	**Publication bias**
**Outcomes^**a**^**	**No. of comparisons**	**Random effect**	***P*-value**	***I^**2**^***	***P*-value**	**Random effect**	**Egger**
C12:0	26	−3.764	<0.001	95.209	<0.001	−0.989	0.001
		(−4.691, −2.838)				(−1.189, −0.790)	
C14:0	26	−3.044	<0.001	96.245	<0.001	−1.168	<0.001
		(−4.021, −2.067)				(−1.436, −0.900)	
C16:0	26	−3.524	<0.001	95.774	<0.001	−3.430	<0.001
		(−4.468, 2.579)				(−4.382, −2.478)	
C18:0	26	2.949	<0.001	96.461	<0.001	1.699	<0.001
		(1.923, 3.976)				(1.220, 2.178)	
C18:1 t-11	21	6.336	<0.001	96.290	<0.001	2.322	<0.001
		(5.039, 7.632)				(1.782, 2.862)	
C18:1 c-9	26	3.537	<0.001	96.123	<0.001	1.907	<0.001
		(2.556, 4.517)				(1.663, 2.151)	
C18:2 c-9, t-11	26	4.668	<0.001	95.683	<0.001	0.769	<0.001
		(3.690, 5.646)				(0.577, 0.961)	
C18:2 n-6	28	1.320	<0.001	91.515	<0.001	0.355	0.226
		(0.758, 1.883)				(0.199, 0.512)	
C18:3 n-3	28	2.713	<0.001	98.992	<0.001	0.408	0.189
		(1.651, 3.775)				(0.254, 0.562)	
ΣSFA	28	−3.037	<0.001	96.576	<0.001	−5.104	0.013
		(−4.005, −2.068)				(−7.214, −2.993)	
ΣMUFA	23	0.999	0.108	97.320	<0.001	2.115	0.642
		(−0.220, 2.218)				(−1.291, 5.520)	
ΣPUFA	25	3.104	<0.001	94.561	<0.001	1.885	<0.001
		(2.352, 3.857)				(1.363, 2.407)	

a SFA, saturated fatty acids; MUFA, monounsaturated fatty acids; PUFA, polyunsaturated fatty acids;

b SMD, standardized mean difference;

c Cl, confidence interval;

d *RMD, raw mean difference*.

**Figure 4:**
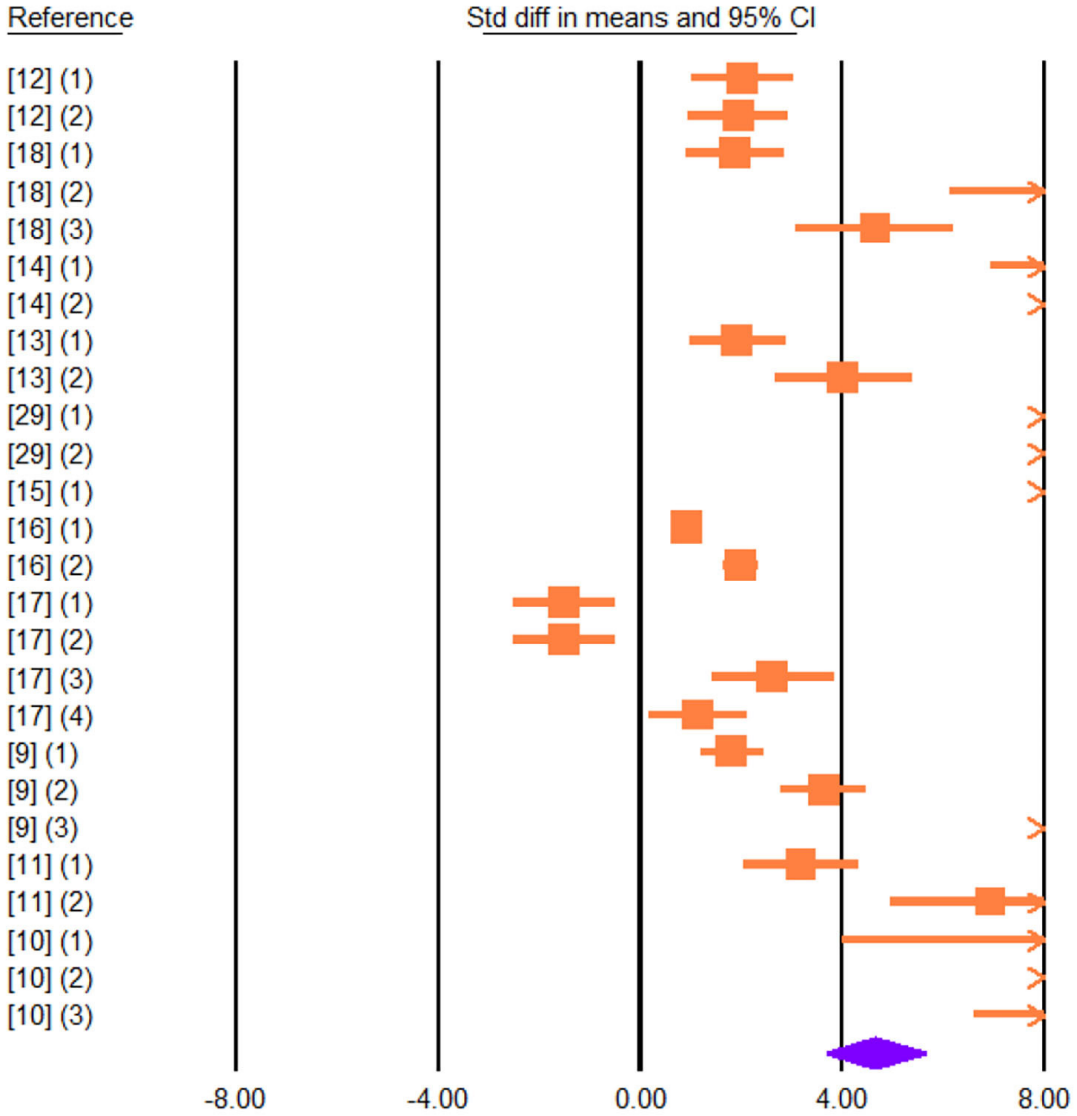
Forest plot of the effect of dietary vegetable sources rich in unsaturated fatty acids on cheese C18:2 c-9, t-11 in dairy ewe based on standardized mean differences (Std. diff in means). The diamond at the bottom indicates the mean effect size, calculated according to a random-effect model. The size of the squares illustrates the weight of each study relative to the mean effect size. Smaller squares represent less weight. The horizontal bars represent the 95% confidence intervals for the study.

Adding vegetable sources rich in UFA in the diet led to an increase in the concentration of C18:1 t-11 (SMD = 6.336; *P* < 0.001), C18:1 c-9 (SMD = 3.537; *P* < 0.001), C18:2 c-9, t-11 (SMD = 4.668; *P* < 0.001; Figure 4), C18:2 n-6 (SMD = 1.320; *P* < 0.001), and C18:3 n-3 (SMD = 2.713; *P* < 0.001) in cheese.

**Figure 5:**
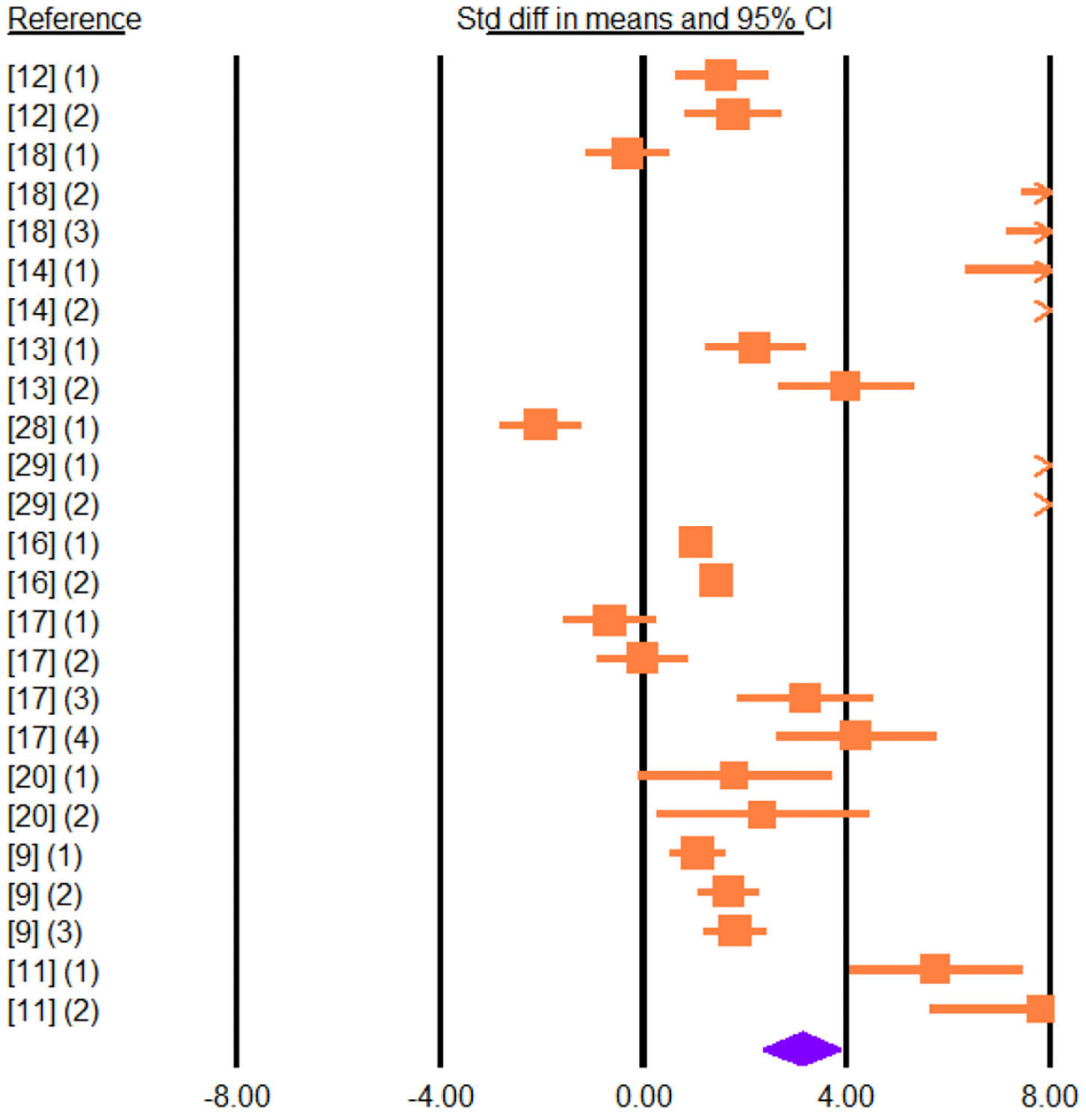
Forest plot of the effect of dietary vegetable sources rich in unsaturated fatty acids on cheese total polyunsaturated fatty acids in dairy ewe based on standardized mean differences (Std. diff in means). The diamond at the bottom indicates the mean effect size, calculated according to a random effects model. The size of the squares illustrates the weight of each study relative to the mean effect size. Smaller squares represent less weight. The horizontal bars represent the 95% confidence intervals for the study.

Vegetable sources rich in UFA in the diet led to decrease in the concentration of total saturated FA (SFA; SMD = −3.037; *P* < 0.001) and increased total PUFA (SMD = 3.104; *P* < 0.001; Figure 5) while total monounsaturated FA (MUFA) in cheese did not increase (SMD = 0.999; *P* = 0.108).

Heterogeneity of FA was significant (*I*^2^, Table 5). C12:0, C14:0, C16:0, C18:0, C18:1 t-11, C18:1 c-9, C18:2 c-9, t-11, C18:2 n-6, C18:3 n-3, and MUFA showed that ripening time was significant cause of heterogeneity between studies, whereas for SFA and PUFA ripening time was not significant (Table 6). In other words, these results showed that with increasing ripening time, the concentrations of C12:0, C14:0, and C16:0 increased and the concentrations of C18:0, C18:1 t-11, C18:1 c-9, C18:2 c-9, t-11, C18:2 n-6, C18:3 n-3, and MUFA decreased.

**Table 6:** Summary of meta–regression analysis output for cheese fatty acids that were influenced by ripening time.

**Fatty acid**	**Item^**a**^**	**Coefficient**	**95% CI^**b**^**	***P*-value**
C12:0	Intercept	−6.316	−7.421, −5.210	<0.001
	Ripening time	0.052	0.036, 0.068	<0.001
C14:0	Intercept	−5.504	−6.720, −4.288	<0.001
	Ripening time	0.050	0.032, 0.068	<0.001
C16:0	Intercept	−5.503	−6.797, −4.210	<0.001
	Ripening time	0.040	0.021, 0.060	<0.001
C18:0	Intercept	8.264	6.851, 9.678	<0.001
	Ripening time	−0.100	−0.120, −0.079	<0.001
C18:1 t-11	Intercept	13.041	10.611, 15.471	<0.001
	Ripening time	−0.159	−0.208, −0.111	<0.001
C18:1 c-9	Intercept	5.652	4.411, 6.894	<0.001
	Ripening time	−0.045	−0.063, −0.027	<0.001
C18:2 c-9, t-11	Intercept	6.694	5.196, 8.191	<0.001
	Ripening time	−0.040	−0.063, −0.018	<0.001
C18:2 n-6	Intercept	1.972	1.098, 2.847	<0.001
	Ripening time	−0.014	−0.027, −0.001	0.027
C18:3 n-3	Intercept	3.971	2.400, 5.541	<0.001
	Ripening time	−0.026	−0.051, −0.002	0.032
ΣSFA	Intercept	−2.851	−4.317, −1.386	<0.001
	Ripening time	−0.003	−0.026, 0.019	0.771
ΣMUFA	Intercept	−2.488	−4.634, −0.343	0.023
	Ripening time	0.060	0.030, 0.091	<0.001
ΣPUFA	Intercept	2.993	1.729, 4.258	<0.001
	Ripening time	0.002	−0.016, 0.022	0.767

a SFA, saturated fatty acids; MUFA, monounsaturated fatty acids; PUFA, polyunsaturated fatty acids;

b *CI, confidence interval*.

**Figure 6:**
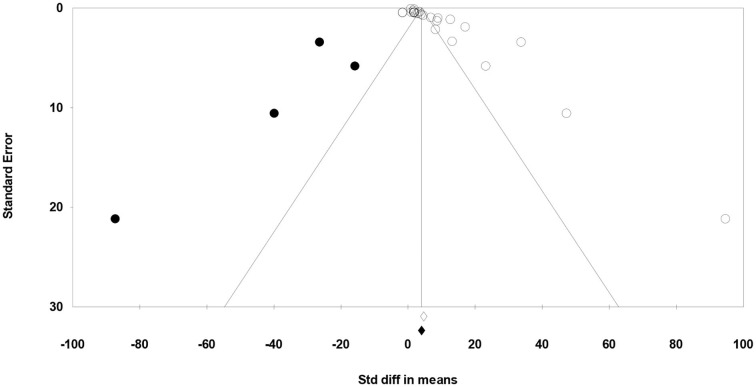
Funnel plot of the standardized mean difference (Std. diff in means) of studies (empty circles) from all studies with C18:2 c-9, t-11. The solid dots are the potentially missing studies imputed from the trim-and-fill method. The open diamond represents the mean and confidence interval of the existing studies, and the solid diamond represents the mean and confidence interval if the theoretically imputed studies were included in the meta-analysis.

**Figure 7:**
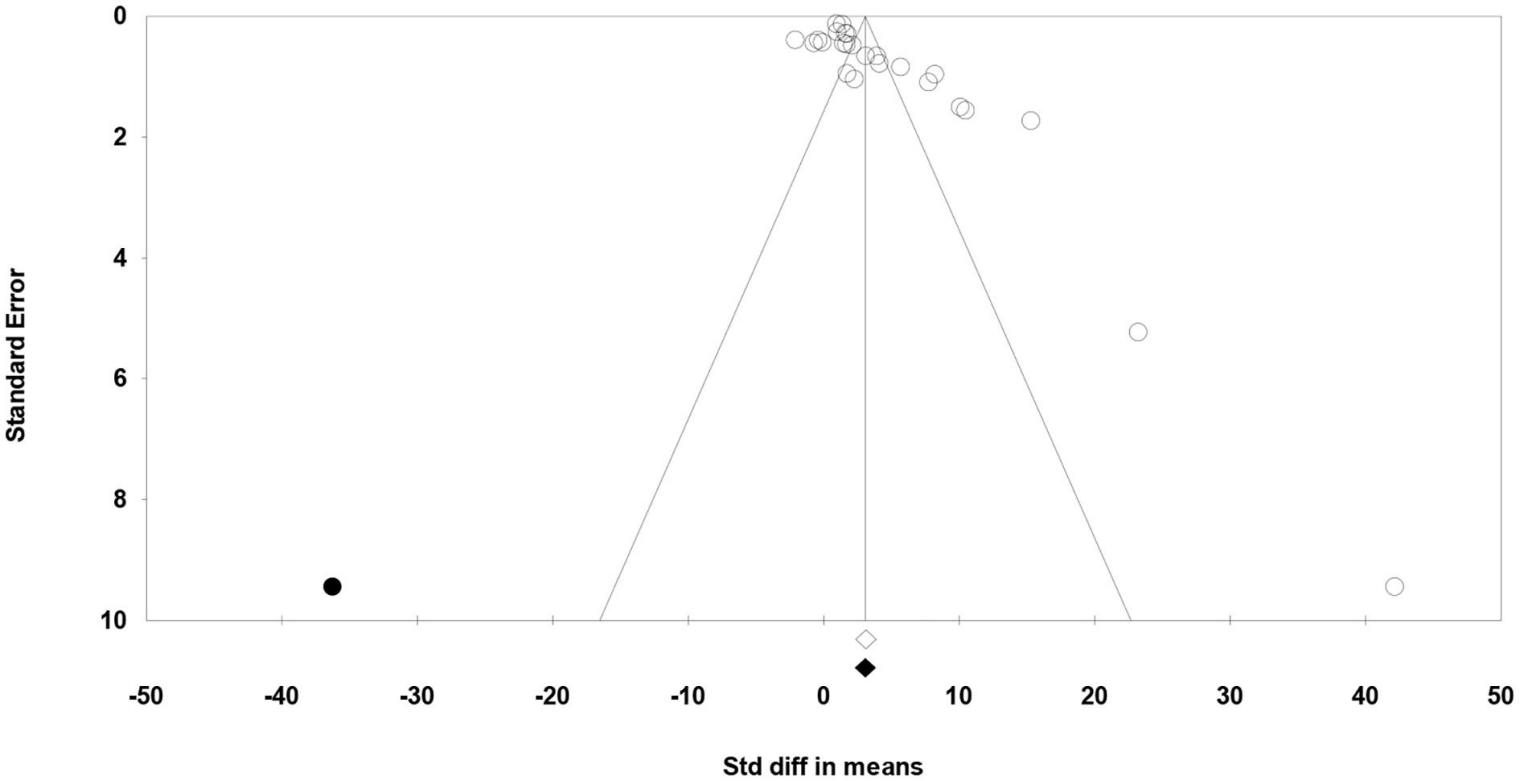
Funnel plot of the standardized mean difference (Std. diff in means) of studies (empty circles) from all studies with polyunsaturated fatty acids. The solid dots are the potentially missing studies imputed from the trim-and-fill method. The open diamond represents the mean and confidence interval of the existing studies, and the solid diamond represents the mean and confidence interval if the theoretically imputed studies were included in the meta-analysis.

Meta-regression suggested that the feeding system (preserved roughages vs. pasture) was not an important factor contributing to the heterogeneity observed in C18:2 c-9, t-11, C18:2 n-6, C18:3 n-3, SFA, MUFA, and PUFA (data not shown). Egger's test showed no publication bias for C18:2 n-6, C18:3 n-3, and MUFA, while a publication bias was detected for other fatty acids (*P* < 0.1; Table 5). For example, publication bias for C18:2 c-9, t-11, and PUFA is shown by four missing observations and one missing observation to the left of the funnel plots, respectively (Figures 6, 7).

## Discussion

In this meta-analysis, supplementation of vegetable sources rich in UFA did not affect MY, MFP, and MPP. It is known that supplementing fat in dairy ruminant diets is a nutritional strategy to increase dietary energy density in order to sustain milk production (30). However, differences among dairy ruminant species have been reported in relation to the energy partitioning consequent to dietary fat supplementation (31). However, the overall effect of dietary fat on MY is a result of a coordinated metabolic process where variables such as sources of fat, type of fat, level of inclusion in the diet, and stage of lactation play an important role (32). Moreover, in the experimental trials included in the meta-analysis, the supplemented and unsupplemented diets were usually isoenergetic. Consequently, the diets differed only for the energy source (fat vs. starch) and not for the energy density. Another key point is that the amount of supplemental fat will be important to improve or maintain dry matter intake and MY without compromising ruminal function (33).

With regard to milk fat content, the effects of supplemental fats were inconsistent. In general, it is known that when ruminants are fed with increasing contents of dietary PUFA and/or specific feeding regimens (such as high concentrate:forage ratio), this leads to inhibition of *de novo* synthesis of milk fat, which might lead to decreases in milk fat contents (7, 34). According to the biohydrogenation theory proposed by Bauman and Griinari (35), “under certain dietary conditions the pathways of rumen biohydrogenation are altered to produce unique fatty acid intermediates, which are potent inhibitors of milk fat synthesis.” Subsequent studies highlighted that rumen biohydrogenation intermediates (such as C18:2 t-10, c-12) of C18:2 and C18:3 FA (36) are able to interfere with lipid metabolism. Those changes in lipid metabolism can induce differential expression of lipid-related genes at adipose tissue (37, 38) and at mammary gland (39, 40) levels in ruminants. However, in the studies considered in the present meta-analysis, the fat supplementation did not exceed 6% of DM intake and it was not associated with high-concentrate feeding regimes and, thus, limited the risk for milk fat depression (41).

The lack of effects of feeding vegetable sources rich in UFA on milk protein contents in sheep was expected as the studies that were analyzed in this meta-analysis adopted a safe dose of lipid supplementation, in relation to the potential effect of dietary lipids on rumen metabolism. In ruminants, milk protein contents can be affected when dietary fermentable carbohydrates are replaced with fat, and this results in decreases of rumen microbial protein yields and increases in the use of amino acids for gluconeogenesis (42). Since milk protein content was not affected by dietary treatments, we can suppose that the range of lipid supplementation adopted in the experiment considered was not detrimental for the rumen fermentation of dietary carbohydrates.

The meta-regression results indicated that DIM influences MFP and MPP heterogeneity. This suggests that with increasing DIM, the MFP and MPP increase in animals receiving vegetable sources rich in UFA. These results should be interpreted with caution because they were based on very few studies.

The main emphasis of this meta-analysis was to quantify changes on cheese FA profile in sheep fed vegetable sources rich in UFA. In terms of effect size, the overall effect of lipid supplementation on cheese FA composition was the increment of PUFA and decrease of SFA content. Interestingly, the magnitude of the effect size based on SMD is similar for PUFA and SFA (+3.104 and −3.037 for PUFA and SFA, respectively). This result is in agreement with previous data reported for dairy cattle and goats (43). The magnitude of the effect size was particularly relevant for C18:2 c-9, t-11, C18:1 t-11, and C18:0, as a consequence of the common biohydrogenation pathway shared by the different UFA (C18:3 n-3, C18:2 n-6, C18:1 c-9) present in the dietary supplements. In fact, under normal rumen function (when there is not trans-10 shift) and irrespective to the lipid supplement included in the diet, C18:1 t-11 and C18:0 are the main products of the rumen biohydrogenation process (44). The C18:2 c-9 t-11 isomer is strictly linked to the availability of C18:1 t-11, being mainly produced in the mammary gland by way of the delta-9 desaturase enzyme (7, 34). Interestingly, the average amount of C18:1 c-9 was significantly increased by lipid supplementation, irrespective to the source of dietary lipids. Taking into consideration the studies considered in the present meta-analysis, only 3 out of 14 included a dietary source of C18:1 c-9 (olive oil or olive cake), whereas the other lipid sources provided mainly C18:2 n-6 or C18:3 n-3 FA. In addition, in this meta-analysis, publication bias was observed for cheese FA profile other than C18:2 n-6, C18:3 n-3, and MUFA. One of the problems that we faced in this meta-analysis is the fact that small trials or studies with negative results tend not to be published and that limits the amount of relevant studies accounted for the analysis (26, 27).

The meta-regression results indicated that ripening time influences cheese FA profile heterogeneity. The ripening time is from 1 to 120 days between studies, and ripening time may be one of the factors affecting the concentration of FA in cheese. These results should be interpreted with caution because they were based on a small number of studies. At this point, it is difficult to find a solid explanation for the relation between ripening time and cheese FA profiles. The studies that were analyzed have different cheese manufacturing protocols. Each study used different starter cultures and/or enzymes and thus lipolytic and esterolytic activities (45) may vary between each type of cheese. This issue warrants further attention.

It is important to note that duodenal flow of C18:0 has been positively related to the amount of dietary unsaturated FA (46); thus, the overall effect of dietary lipid supplementation on the content of C18:1 c-9 in milk and cheese was probably due to the conversion of C18:0 into C18:1 c-9 by mammary delta-9 desaturase enzyme. In fact, in dairy cows, Glasser et al. (47) demonstrated that mammary desaturation of C18:0 into C18:1 c-9 was directly proportional to the mammary uptake of the substrate.

On average, the effect size of the dietary lipid supplementation on C18:3 n-3 content in cheese fat was comparable to that obtained for C18:2 n-6, although the number of studies including C18:3 n-3 dietary sources was larger. In this sense, caution must be paid when interpreting our results as 12 out of 14 studies included in the meta-analysis used dietary treatments with linseed or flaxseed as lipid source. Our data shows that there are indeed changes toward healthier sheep cheeses from a human standpoint, as there are increases in some bioactive FA such as C18:1 c-9, which have been reported to inhibit the cycle of cancer cells, leukemia, and induction of apoptosis in experimental models (48). Our data also showed that some *trans* FA such as C18:1 t-11, C18:2 c-9, t-11, and C18:3 n-3 increase in sheep cheeses when sheep are supplemented with vegetable UFA. In humans, these natural *trans* FA have been related to positive effects on inflammation, obesity, and type 2 diabetes (49). More specifically, clinical trials demonstrated that naturally conjugated linoleic acid (CLA; C18:2 c-9, t-11) and C18:3 n-3-enriched cheeses are able to ameliorate the plasma lipid profile, and to reduce endocannabinoid biosynthesis (50), to reduce plasma inflammatory markers, such as IL-6, IL-8, and TNF-a (51). Moreover, feeding CLA-enriched cheeses from different sources (bovine, ovine, and caprine) for 2 months improved in humans the n-3 PUFA score, by increasing plasma docosahexaenoic acid, and the effect was proportional to the CLA content in the cheese (52).

From a more practical perspective, it is important to note that the feeding system (preserved roughages vs. pasture) was not an important factor contributing to the heterogeneity observed in C18:2 c-9, t-11, C18:2 n-6, C18:3 n-3, SFA, MUFA, and PUFA. It is difficult to make a robust interpretation from these results. When the selected studies were revised, we found out that some of them were either focused on animal production while others were focused only on cheese production; therefore, details on diet and feeding regimes were not always reported. In addition, this issue did not allow us to estimate forage-to-concentrate ratios.

### Reflections and Projections

Our meta-analysis has shown that for the past two decades efforts have been made to improve sheep cheese FA profile, and our data suggest that the use of vegetable sources is a feasible strategy. However, one of the missing details in all the analyzed scientific reports is the lack of cost–effect analysis. The economic value of milk fat and its responsiveness to feeding management strategies provides strong interest in maximizing milk fat production (53), and its relation to cheese manufacturing is very important. High contents of milk fat are related to increases in cheese yield due to higher recovery of milk nutrients in the curd (54). Thus, an economic analysis is needed in order to provide recommendations that are more balanced to farmers and dairy industry.

One of the problems that we faced when analyzing the available data was that not all studies report or use the data set framework and, thus, the chemical composition of diet and dietary FA profiles was not found in many cases. On the one hand, this limitation is due to that we could not use some parameters as covariates in meta-regression. On the other hand, in biological terms, it is difficult to obtain a complete “picture” of some studies as the input–output analysis was not always reported, and it would be very important to fully understand the effects of ruminant diet on the nutritional quality of cheeses.

## Conclusions

Overall, our meta-analysis data indicated that dietary vegetable sources rich in UFA reduces total saturated FA while increasing contents of potential healthy FA such as natural trans (C18:1 t-11 and C18:2 c-9, t-11) and PUFA (C18:2 and C18:3) in cheese without detrimental effects on milk yield and milk composition.

## Data Availability Statement

The raw data supporting the conclusions of this article will be made available by the authors, without undue reservation.

## Author Contributions

EV-B-P and MG-R: conceptualization. EV-B-P, BD, FM, and PG-C: data curation and investigation. FM and BD: formal analysis. BD: methodology and software. EV-B-P: supervision. EV-B-P, BD, and FM: validation. EV-B-P, BD, and MM: visualization. EV-B-P, PG-C, MG-R, and MM: writing—original draft. EV-B-P, BD, FM, PG-C, MG-R, and MM: writing—review & editing. All authors: reviewed and approved the final manuscript.

## Conflict of Interest

The authors declare that the research was conducted in the absence of any commercial or financial relationships that could be construed as a potential conflict of interest.
